# Menopause-Associated Anterior Cutaneous Nerve Entrapment Syndrome: A Case Highlighting Perineural Microenvironmental Pathology

**DOI:** 10.7759/cureus.107006

**Published:** 2026-04-14

**Authors:** Kyeong Deok Lee

**Affiliations:** 1 General Medicine, TMG Asaka Medical Center, Saitama, JPN; 2 Pediatric Surgery, TMG Asaka Medical Center, Saitama, JPN

**Keywords:** acnes, anterior cutaneous nerve entrapment syndrome, chronic abdominal wall pain, estrogen, menopause, peripheral nerve injury (pni), vascular remodeling

## Abstract

Anterior cutaneous nerve entrapment syndrome (ACNES) is an underrecognized cause of abdominal wall pain and is commonly diagnosed based on localized physical findings and response to local anesthetic nerve block. While this approach is clinically practical, it provides limited insight into the underlying pathophysiology and may contribute to diagnostic delay and repeated unnecessary investigations. ACNES represents a form of localized chronic compressive neuropathy characterized by perineural fibrosis and vascular involvement; however, little attention has been paid to systemic conditions that may influence nerve vulnerability or modulate the perineural environment.

We report a case of menopause-associated ACNES in a woman presenting with severe abdominal pain that led to multiple emergency visits and extensive negative visceral evaluations. Definitive diagnosis was established based on physical findings and response to nerve block. Preoperative rehabilitation resulted in partial symptom improvement, followed by surgical resection. Operative findings revealed localized adhesions surrounding the cutaneous nerve.

Histopathological examination demonstrated preservation of intrafascial nerve architecture. In contrast, no clearly identifiable peripheral nerve structure was observed in the extrafascial region. Instead, elongated fibrous structures extended longitudinally through the tissue, lacking normal nerve fascicular organization and surrounded by densely packed, irregularly arranged, curled collagen fibers. In addition, marked vascular wall thickening with severe luminal narrowing was observed, predominantly within the subcutaneous tissue. These layer-specific findings indicate pathological alteration of the perineural microenvironment.

The temporal association with the menopausal transition provides a biologically plausible systemic background for these changes, given the known effects of estrogen deficiency on vascular remodeling, connective tissue metabolism, and pain modulation. This case extends the current understanding of ACNES by highlighting menopause as a potential systemic modifier of disease expression and underscores the limitations of relying solely on physical examination and nerve block response for diagnosis. Integrating systemic background with histopathological insight may help refine current conceptual and diagnostic frameworks for ACNES.

## Introduction

Anterior cutaneous nerve entrapment syndrome (ACNES) is a recognized cause of chronic abdominal wall pain resulting from entrapment of the anterior cutaneous branches of the thoracoabdominal nerves as they pass through the abdominal musculature and fascia [1,2]. Diagnosis is typically based on focal tenderness, a positive Carnett’s sign (increased pain during abdominal muscle contraction), and symptomatic relief following local anesthetic injection [3]. Although this diagnostic framework is effective in identifying the pain source, it largely conceptualizes ACNES as a localized mechanical neuropathy. As a result, little attention has been given to systemic conditions that may influence nerve vulnerability or modulate the perineural environment.

In our previous case series [4], we demonstrated that ACNES represents a form of chronic compressive neuropathy characterized by perineural fibrosis, vascular compromise, and axonal degeneration. Despite these insights, the systemic factors that modify the perineural microenvironment and contribute to symptom manifestation remain poorly understood. Menopause is a systemic physiological transition characterized by estrogen deficiency, which affects vascular remodeling [5-7], connective tissue metabolism [5,8,9], and pain modulation [10-14]. These changes may lower the threshold for clinically significant nerve entrapment, yet their relationship to ACNES has not been previously explored in detail.

Here, we report a menopause-associated case of ACNES with distinctive layer-specific histopathological findings, including marked fibrosis and vascular remodeling. By building upon our prior work, this case further refines the pathophysiological framework of ACNES beyond a purely localized mechanical disorder.

## Case presentation

A 56-year-old woman working as a medical secretary presented with a six-month history of severe, localized abdominal pain. Her medical history included surgery for an appendiceal mucinous neoplasm performed 13 years earlier. She was also receiving hormone replacement therapy with estrogen and progestin for menopausal symptoms. She had no history of hypertension, dyslipidemia, diabetes mellitus, or smoking. The pain was initially associated with urinary frequency and was described as sharp and intense, radiating from the right upper quadrant to the back. Owing to the severity and recurrence of symptoms, she made multiple emergency department visits to a regional general hospital. During this period, she underwent extensive diagnostic evaluation, including three computed tomography (CT) scans. She was evaluated by urology, general surgery, gynecology, and gastroenterology services; however, no visceral or structural abnormalities were identified, and she was repeatedly informed that no significant pathology was present.

As the pain persisted and markedly impaired her quality of life, the patient independently researched possible causes of abdominal pain and suspected ACNES. She subsequently visited a pain clinic. At that time, her Numerical Rating Scale (NRS) pain score was 8/10, and the Ogawa Neuropathic Pain Screening Questionnaire was 15/28 [15]. Physical examination revealed a positive Carnett’s sign, leading to a diagnosis of ACNES. Ultrasonography demonstrated a vascular structure adjacent to the suspected entrapment site to the right of the umbilicus. Local anesthetic nerve block injections were administered five times, resulting in significant but temporary pain relief. The patient was referred to our institution for definitive treatment.

**Figure 1:**
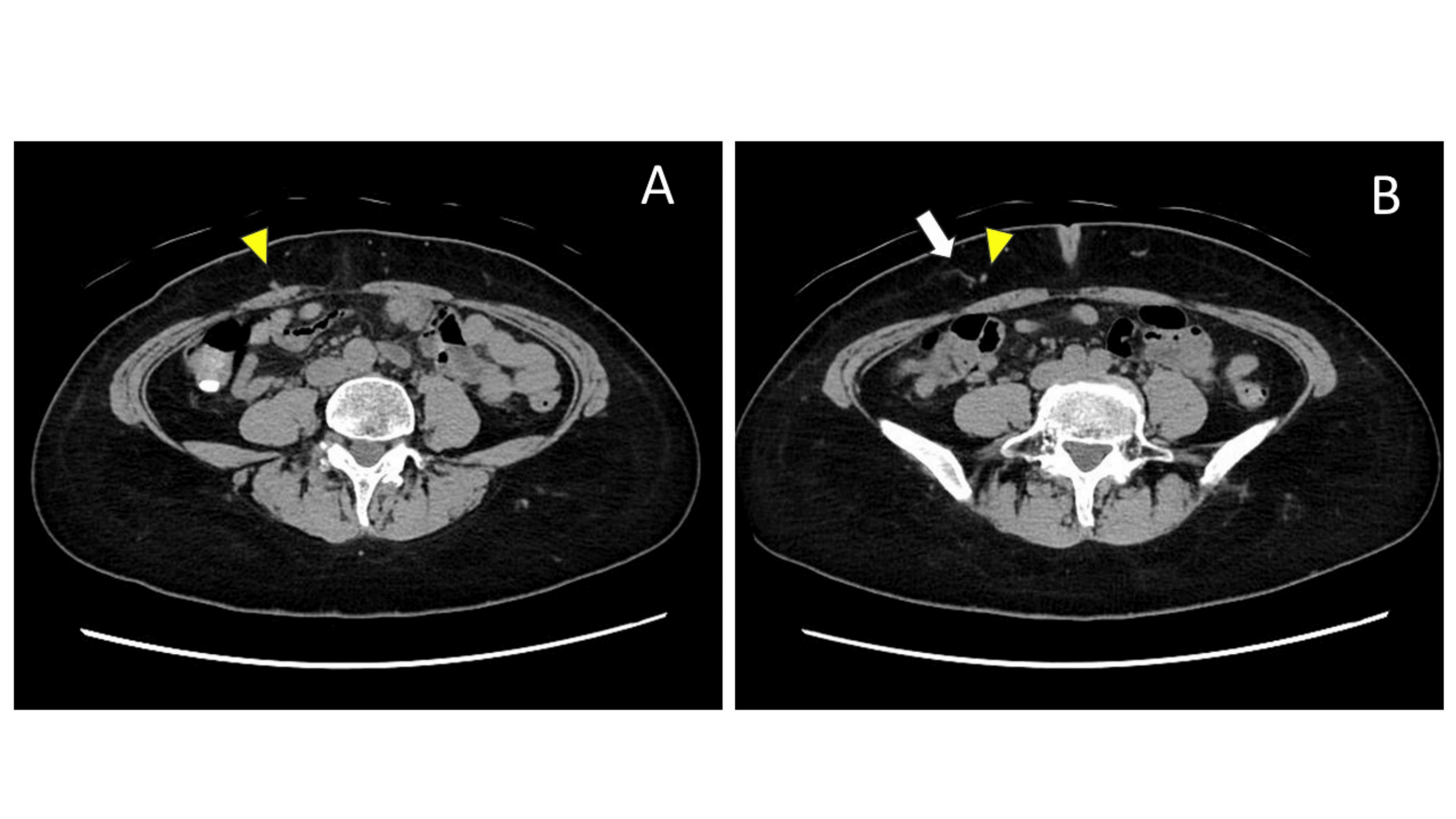
Computed tomography images of the abdomen. (A) An axial CT image obtained at the level of the patient’s maximal tenderness demonstrates a subtle linear structure extending from the rectus abdominis muscle toward the subcutaneous tissue (yellow arrowhead), consistent with the expected course of an anterior cutaneous nerve. (B) An axial image obtained at a slightly more cranial level shows continuity of the linear structure identified in panel A (yellow arrowhead). Immediately adjacent to this structure on the right side, a faint, cord-like density is observed (white arrow), without any definite visceral abnormality or intra-abdominal pathology corresponding to the patient’s localized pain.

At our institution, physical examination again revealed a localized point of maximal tenderness approximately 35 mm to the right of the umbilicus, with pain exacerbated by abdominal muscle contraction. CT imaging, which was performed at the regional general hospital, had already demonstrated a cutaneous nerve corresponding to the area of maximal tenderness (Figure 1). Preoperative rehabilitation focusing on trunk mobility and abdominal wall conditioning resulted in partial symptom improvement (NRS pain score improved from 8/10 to 5/10) but did not achieve complete resolution. Given persistent symptoms, surgical intervention was pursued.

Intraoperatively, a cutaneous nerve corresponding precisely to the preoperative point of maximal tenderness was identified. The nerve was surrounded by adherent fibrous tissue. Sharp resection of the affected nerve segment, together with the surrounding subcutaneous tissue, was performed using surgical scissors. Beneath the fascia, no significant adhesions or fibrosis were observed, and the nerve could be mobilized easily, separating naturally from the underlying tissue.

**Figure 2:**
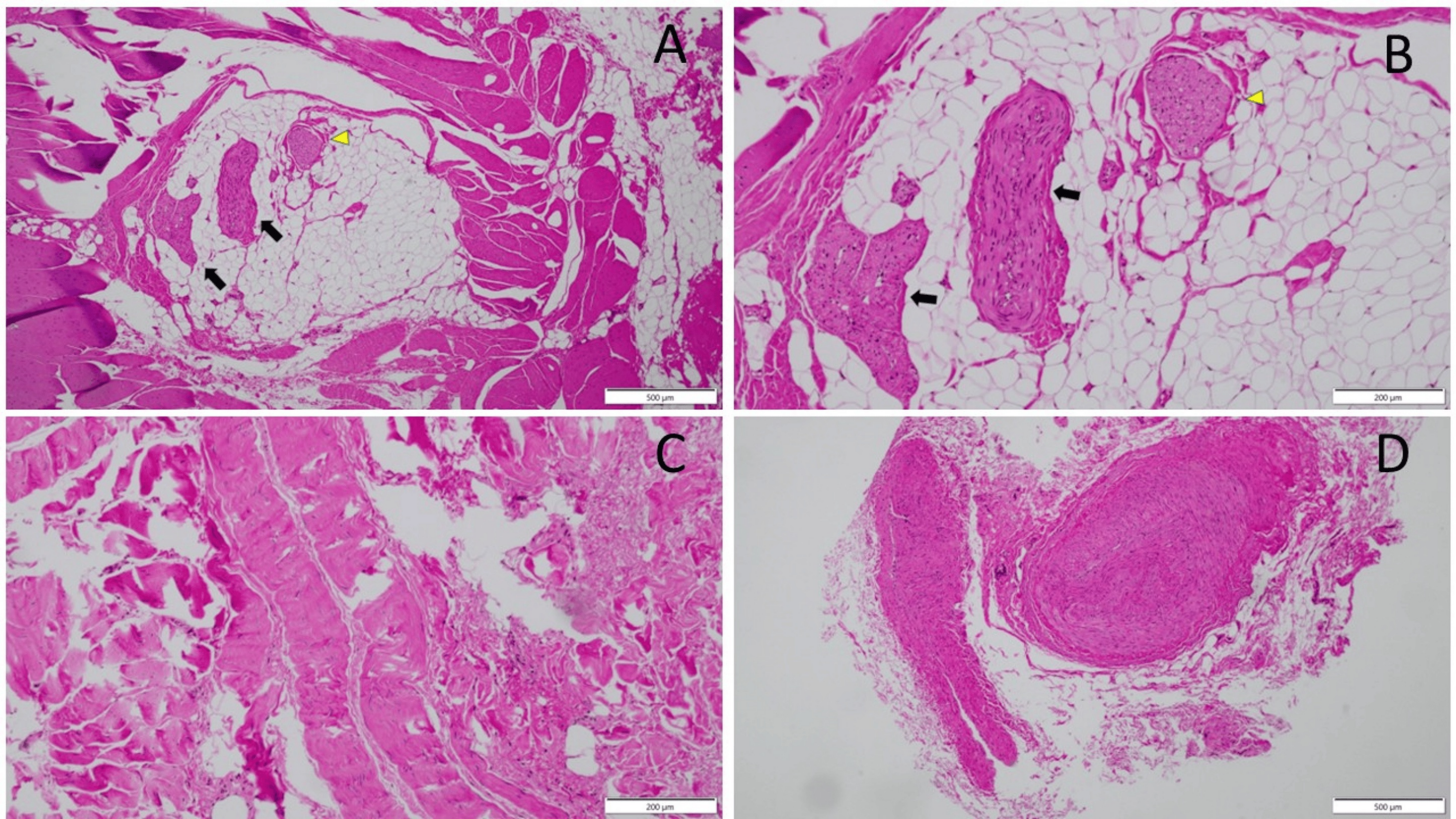
Layer-specific histopathological findings of the resected anterior cutaneous nerve and surrounding tissues (A) A peripheral nerve bundle (yellow arrowhead) and an accompanying blood vessel (black arrows) within intrafascial tissue, surrounded by skeletal muscle and adipose tissue. Scale bar: 500 μm.(B) Higher-magnification view of the intrafascial tissue showing the nerve bundle with only mild perineurial fibrosis. An adjacent blood vessel demonstrates a thickened wall and a narrowed lumen. Adipose tissue is interposed between the nerve bundle and the blood vessel. Scale bar: 200 μm.(C) Section from the extrafascial structure showing marked collagen deposition and disorganized fibrous architecture without identifiable nerve elements, consistent with advanced fibrotic change. Scale bar: 200 μm.(D) Cross-section of the surrounding subcutaneous tissue showing pronounced vascular wall thickening with a markedly narrowed vascular lumen. Scale bar: 500 μm.

Within the fascial layer, a peripheral nerve bundle was identified with largely preserved architecture (Figure 2A). The nerve bundle showed no evidence of axonal degeneration, demyelination, or inflammatory infiltration and was surrounded by soft adipose tissue. In the same intrafascial region, two adjacent vessels exhibited wall thickening with luminal narrowing. No inflammatory or atherosclerotic changes were noted (Figure 2B).

In the extrafascial region, a clearly identifiable peripheral nerve structure was absent. Instead, elongated fibrous structures extended longitudinally through the tissue. These structures lacked normal nerve fascicular organization and were surrounded by densely packed, irregularly arranged, curled collagen fibers (Figure 2C).

In the surrounding subcutaneous tissue, multiple medium-sized vessels demonstrated abnormal vascular remodeling, characterized by disproportionately thickened walls and severely narrowed lumina. In several vessels, intraluminal blood cells were scarcely identifiable. No significant perivascular inflammatory infiltrates were observed (Figure 2D).

The postoperative course was largely uneventful. The patient experienced rapid and near-complete resolution of the abdominal pain immediately after surgery. Mild residual pain in the right hypochondriac region, distinct from the surgical wound, persisted but did not warrant further surgical intervention and has been managed with periodic nerve block therapy at a pain clinic. Delayed wound healing with postoperative seroma formation was observed, which was managed conservatively without further complications. No recurrence of the primary pain was observed, and both the patient and the surgeon were satisfied with the overall outcome.

## Discussion

To our knowledge, this is the first report to suggest an association between menopause and ACNES, highlighting the potential role of the perineural microenvironment in disease expression. These findings may help refine the diagnostic framework and inform therapeutic strategies for ACNES.

ACNES is typically diagnosed using localized physical findings and confirmation by response to local anesthetic nerve block [2,3]. This pragmatic approach is effective in identifying abdominal wall pain as non-visceral in origin; however, it provides limited insight into why symptoms emerge in certain patients and not others. The present case illustrates how reliance on this framework alone may contribute to diagnostic delay and repeated, potentially unnecessary investigations. In contrast, our previous work and the current study emphasize a broader, mechanism-oriented approach to ACNES management, integrating radiological findings, rehabilitation strategies, surgical findings, and histopathological evaluation [4]. By examining structural and microenvironmental changes surrounding the entrapped nerve, this approach aims not only to localize the source of pain but also to clarify its underlying causes, thereby enabling the use of diverse clinical information to guide more targeted and durable pain relief.

In this case, the patient experienced recurrent severe pain leading to multiple emergency visits and underwent CT examinations three times, along with evaluations by several specialties, all of which failed to identify a cause. Such a clinical course is not uncommon in ACNES and reflects a broader diagnostic gap, in which abdominal wall pain is insufficiently contextualized within systemic and functional frameworks [3].

Importantly, this patient’s occupational background as a secretary, involving prolonged sitting and sustained trunk positioning, may have contributed to symptom modulation. While occupation alone cannot be considered a causative factor, repetitive postural loading and reduced trunk mobility may exacerbate vulnerability of the abdominal wall nerves, particularly in the presence of altered tissue compliance.

In our previous studies, we emphasized that an effective treatment strategy for ACNES includes radiological findings, perioperative rehabilitation, surgical intervention, and careful pathological evaluation [4]. Preoperative CT allows accurate identification of the affected nerve, thereby facilitating precise and targeted surgical neurectomy. Preoperative rehabilitation can reduce muscle tension, clarify the localization of pain, and in some cases help determine whether the condition is reversible or has progressed to an irreversible state. We also reported that surgical neurectomy provides valuable information on pathological changes, which may contribute to postoperative treatment strategies and the prevention of recurrence.

In the present case, a CT scan, which was performed at the regional general hospital, had already demonstrated a cutaneous nerve extending from the rectus abdominis muscle into the subcutaneous tissue, suggesting a structural basis for nerve entrapment (Figure 1). The partial symptom improvement observed following preoperative rehabilitation further supports this interpretation. Improvement with rehabilitation suggests that at least part of the pain was mediated by functional and potentially reversible factors, such as muscle tone imbalance or altered fascial dynamics. However, incomplete resolution indicates that structural or microenvironmental pathology persisted, ultimately necessitating surgical intervention.

From a general neuropathological standpoint, peripheral nerve injury can be viewed as a stage-dependent continuum [16-20]. In the acute phase, breakdown of the blood-nerve barrier increases endoneurial vascular permeability, leading to intrafascicular edema [16-20]. Accumulated fluid separates axons, promotes demyelination, and causes partial axonal fragmentation [16,17]. In mechanical or ischemic injury, the perineurial component of the blood-nerve barrier may also be affected, sometimes with focal perineurial discontinuity and spatial displacement of axonal bundles [16]. In the chronic phase, endoneurial edema is gradually reabsorbed and replaced by interstitial fibrosis. Progressive degeneration includes axonal disruption, myelin breakdown with macrophage clearance, and replacement of Schwann cells by collagen-producing fibroblast-like cells [18]. Schwann cells simultaneously undergo phenotypic modulation toward a repair-oriented state, with down-regulation of myelin-related genes and up-regulation of trophic and cytokine pathways. These changes are accompanied by thickening of the epineurium and perineurium, fibrosis of interfascicular tissue, and disorganization of the microvasculature [16-20]. Ultimately, prolonged perineural stress may culminate in loss of identifiable nerve elements, with replacement by dense, disorganized collagenous structures, representing the end-stage morphology of chronic peripheral nerve injury.

The operative and histopathological findings in the present case provide critical insight into this process. Surgically, the affected cutaneous nerve was surrounded by adherent tissue at the site corresponding precisely to the point of maximal tenderness, whereas deeper fascial layers were free of significant adhesion or fibrosis. Histopathological examination demonstrated a clear layer-dependent pattern: within the fascial layer, nerve architecture was largely preserved (Figure 2A, 2B), whereas in the extrafascial region, no identifiable peripheral nerve structures were present. Instead, elongated fibrous strands composed of densely packed, irregularly arranged, curled collagen fibers were observed, consistent with an advanced, end-stage fibrotic transformation of the perineural microenvironment (Figure 2C).

In contrast, the surrounding subcutaneous tissue exhibited prominent vascular pathology. Multiple medium-sized vessels showed pronounced wall thickening with severe luminal narrowing (Figure 2D). These changes occurred in the absence of significant inflammatory infiltration or atherosclerotic features and are morphologically consistent with chronic vascular remodeling. Given the patient’s postmenopausal status, such microvascular alterations may plausibly reflect estrogen-deficiency-associated vascular remodeling, which is known to impair endothelial function, promote smooth muscle proliferation, and induce extracellular matrix accumulation within vessel walls [5,6].

Taken together, these findings suggest that ACNES may arise not solely from intrinsic nerve degeneration but from progressive, layer-dependent remodeling of the perineural microenvironment. In this framework, extrafascial fibrotic transformation represents the terminal stage of chronic nerve injury (Figure 2C), while concurrent estrogen-related microvascular remodeling may exacerbate ischemic vulnerability and mechanical stress on the nerve (Figure 2D), thereby contributing to symptom development and persistence.

Menopausal transition provides a biological background for these changes. Estrogen deficiency is known to impair endothelial function, promote vascular remodeling, and reduce microcirculatory perfusion [5-7]. Peripheral nerves are especially sensitive to such changes [15]. Furthermore, estrogen deficiency alters connective tissue metabolism, favoring fibrosis and reduced fascial elasticity, which may facilitate the formation of localized adhesions [5,8,9]. These systemic effects may lower the threshold at which mechanical or postural factors translate into clinically significant neuropathic pain.

Estrogen deficiency is associated with impaired endothelial function through reduced activity of endothelial nitric oxide synthase and increased oxidative stress, leading to diminished nitric oxide bioavailability and loss of endothelium-dependent vasodilation [6,7]. As a consequence, microvascular beds exhibit reduced adaptive responsiveness to mechanical or metabolic demands. In parallel, estrogen deficiency promotes vascular remodeling by inducing endothelial dysfunction and oxidative stress, which lead to intimal thickening and extracellular matrix alterations [7]. This process results in structurally stiffened microvessels with narrowed luminal caliber. The combined effects of endothelial dysfunction and structural remodeling ultimately reduce microcirculatory perfusion at the capillary level, characterized by decreased flow velocity and impaired oxygen diffusion. Because peripheral nerves have high metabolic requirements and limited tolerance to ischemia, even modest and sustained reductions in microvascular perfusion may alter the perineural microenvironment, favoring chronic hypoxia, fibrotic tissue responses, and increased vulnerability to neuropathic pain [16].

Estrogen plays a key role in maintaining connective tissue homeostasis by regulating fibroblast activity [5,8,9]. Estrogen deficiency may shift this balance toward increased collagen deposition, resulting in the accumulation of mechanically stiff collagen and decreased fascial elasticity. These changes may limit the ability of fascial tissue to adapt to normal movement, promoting focal stiffness and adhesion formation around anatomically vulnerable structures. Such altered connective tissue mechanics may facilitate the development of localized adhesions that mechanically constrain peripheral nerve fibers and contribute to persistent pain generation.

In addition to its vascular and connective tissue effects, estrogen plays an important modulatory role in pain processing at both peripheral and central levels [10-14]. At the peripheral level, estrogen influences nociceptor excitability and inflammatory signaling, while at the central level, it modulates synaptic transmission and descending inhibitory pathways involved in pain control [10-13]. Reduced estrogen levels have been associated with increased pain sensitivity and diminished endogenous pain inhibition, potentially amplifying nociceptive input arising from compromised perineural environments [14]. Taken together, these effects provide a biologically plausible framework for understanding why neuropathic pain may become clinically manifest during the menopausal transition, even in the absence of overt intrinsic nerve degeneration.

Furthermore, histopathological findings can transform otherwise invisible pain into visible, objective evidence, alleviating patients’ psychological distress and contributing to high postoperative satisfaction.

Although histopathological evaluation is not required for the diagnosis of ACNES, the present case demonstrates its value in elucidating disease mechanisms that are not apparent from clinical criteria alone. This case does not establish a causal relationship between menopause and ACNES; rather, it proposes a pathophysiologically plausible association supported by clinical course, surgical findings, and pathology.

By integrating systemic physiological factors, functional response to rehabilitation, and detailed pathological findings, this case encourages a broader conceptualization of ACNES. ACNES may represent a condition emerging not only from a purely localized mechanical neuropathy, but from the systemic alterations of the perineural environment.

## Conclusions

ACNES is traditionally diagnosed based on localized physical findings and response to nerve block injection. However, this case demonstrates that such an approach may be insufficient to fully explain the underlying disease mechanism.

Building on our previous work demonstrating chronic compressive neuropathy as a pathological substrate of ACNES, this case further highlights the importance of systemic modifiers in shaping disease expression. Menopause-related vascular and connective tissue changes may contribute to perineural microenvironmental deterioration, leading to fibrosis and functional nerve entrapment. These findings underscore the limitations of diagnosing ACNES solely based on physical examination and nerve block response and support a more comprehensive framework integrating systemic background, imaging, rehabilitation response, and histopathological insight.
